# Genetics of familial adult myoclonus epilepsy: From linkage studies to noncoding repeat expansions

**DOI:** 10.1111/epi.17610

**Published:** 2023-04-17

**Authors:** Mark A. Corbett, Christel Depienne, Liana Veneziano, Karl Martin Klein, Francesco Brancati, Renzo Guerrini, Federico Zara, Shoji Tsuji, Jozef Gecz

**Affiliations:** ^1^ Robinson Research Institute and Adelaide Medical School University of Adelaide Adelaide South Australia Australia; ^2^ Institute of Human Genetics University Hospital Essen, University Duisburg–Essen Essen Germany; ^3^ Institute of Translational Pharmacology National Research Council Rome Italy; ^4^ Departments of Clinical Neurosciences, Medical Genetics, and Community Health Sciences, Hotchkiss Brain Institute and Alberta Children's Hospital Research Institute, Cumming School of Medicine, University of Calgary Calgary Alberta Canada; ^5^ Epilepsy Center Frankfurt Rhine–Main, Department of Neurology, Center of Neurology and Neurosurgery Center for Personalized Translational Epilepsy Research, University Hospital, Goethe University Frankfurt Frankfurt am Main Germany; ^6^ Medical Genetics, Department of Life, Health, and Environmental Sciences University of L'Aquila L'Aquila Italy; ^7^ Laboratory of Human Functional Genomics Istituto di Ricovero e Cura a Carattere Scientifico (IRCCS) San Raffaele Rome Italy; ^8^ Neuroscience and Neurogenetics Department Meyer Children's Hospital Florence Italy; ^9^ Laboratory of Neurogenetics IRCCS Institute "G. Gaslini" Genoa Italy; ^10^ Department of Neurology, Graduate School of Medicine University of Tokyo Tokyo Japan; ^11^ Institute of Medical Genomics International University of Health and Welfare Chiba Japan; ^12^ South Australian Health and Medical Research Institute Adelaide South Australia Australia

**Keywords:** DNA sequencing, molecular genetics, repeat expansion disorders, seizure

## Abstract

Familial adult myoclonus epilepsy (FAME) is a genetic epilepsy syndrome that for many years has resisted understanding of its underlying molecular cause. This review covers the history of FAME genetic studies worldwide, starting with linkage and culminating in the discovery of noncoding TTTTA and inserted TTTCA pentanucleotide repeat expansions within six different genes to date (*SAMD12*, *STARD7*, *MARCHF6*, *YEATS2*, *TNRC6A*, and *RAPGEF2*). FAME occurs worldwide; however, repeat expansions in particular genes have regional geographical distributions. FAME repeat expansions are dynamic in nature, changing in length and structure within germline and somatic tissues. This variation poses challenges for molecular diagnosis such that molecular methods used to identify FAME repeat expansions typically require a trade‐off between cost and efficiency. A rigorous evaluation of the sensitivity and specificity of each molecular approach remains to be performed. The origin of FAME repeat expansions and the genetic and environmental factors that modulate repeat variability are not well defined. Longer repeats and particular arrangements of the TTTTA and TTTCA motifs within an expansion are correlated with earlier onset and increased severity of disease. Other factors such as maternal or paternal inheritance, parental age, and repeat length alone have been suggested to influence repeat variation; however, further research is required to confirm this. The history of FAME genetics to the present is a chronicle of perseverance and predominantly collaborative efforts that yielded a successful outcome. The discovery of FAME repeats will spark progress toward a deeper understanding of the molecular pathogenesis of FAME, discovery of new loci, and development of cell and animal models.

**Key Points**

- There are six independent genes implicated in FAME
- FAME is caused by a noncoding intronic TTTTA and an inserted TTTCA pentamer repeat expansion in one of these six genes
- Longer repeat expansions are correlated with earlier disease onset and greater disease severity
- FAME repeat expansions show somatic and germline instability

## 1 INTRODUCTION

Familial adult onset myoclonus epilepsy (FAME) is a rare, autosomal dominant disorder characterized by tremorlike cortical myoclonus in the second to third decade of life and infrequent generalized tonic–clonic and/or myoclonic seizures that usually start later.^1^ Cortical myoclonus in FAME is identified by giant somatosensory evoked potentials, long latency reflex, and cortical spikes preceding myoclonus based on jerk‐locked averaging of electrophysiological measures.^1^ The definition of FAME as a unique epilepsy syndrome was critical in identifying large families for genetic studies and the eventual discovery of the noncoding TTTTA and inserted TTTCA repeat expansions that cause FAME.

## 2 EARLY LINKAGE STUDIES AND THE IDENTIFICATION OF MULTIPLE FAME LOCI

A genetic marker is defined as a site in the genome with a known location. These markers are usually sites within the human genome of frequent sequence variation in the general population. Markers that segregate with disease in a family are likely to be physically adjacent to the genetic cause of the disease in the genome. Genetic linkage analysis, where multiple markers are used to hunt down the location of a disease‐causing variant, was instrumental in early genetic investigations of FAME.

The first reported linkage study of FAME (or benign adult familial myoclonus epilepsy) targeted the gene for dentatorubral pallidoluysian atrophy (*DRPLA*), in which a causative CAG repeat expansion had been discovered, and the *GABRB1*, *GABRB3*, and *GABRB6* genes.^2^ In all cases, there was no linkage to these genes in a large Japanese family, suggesting that FAME was a distinct genetic epilepsy syndrome.

**FIGURE 1:**
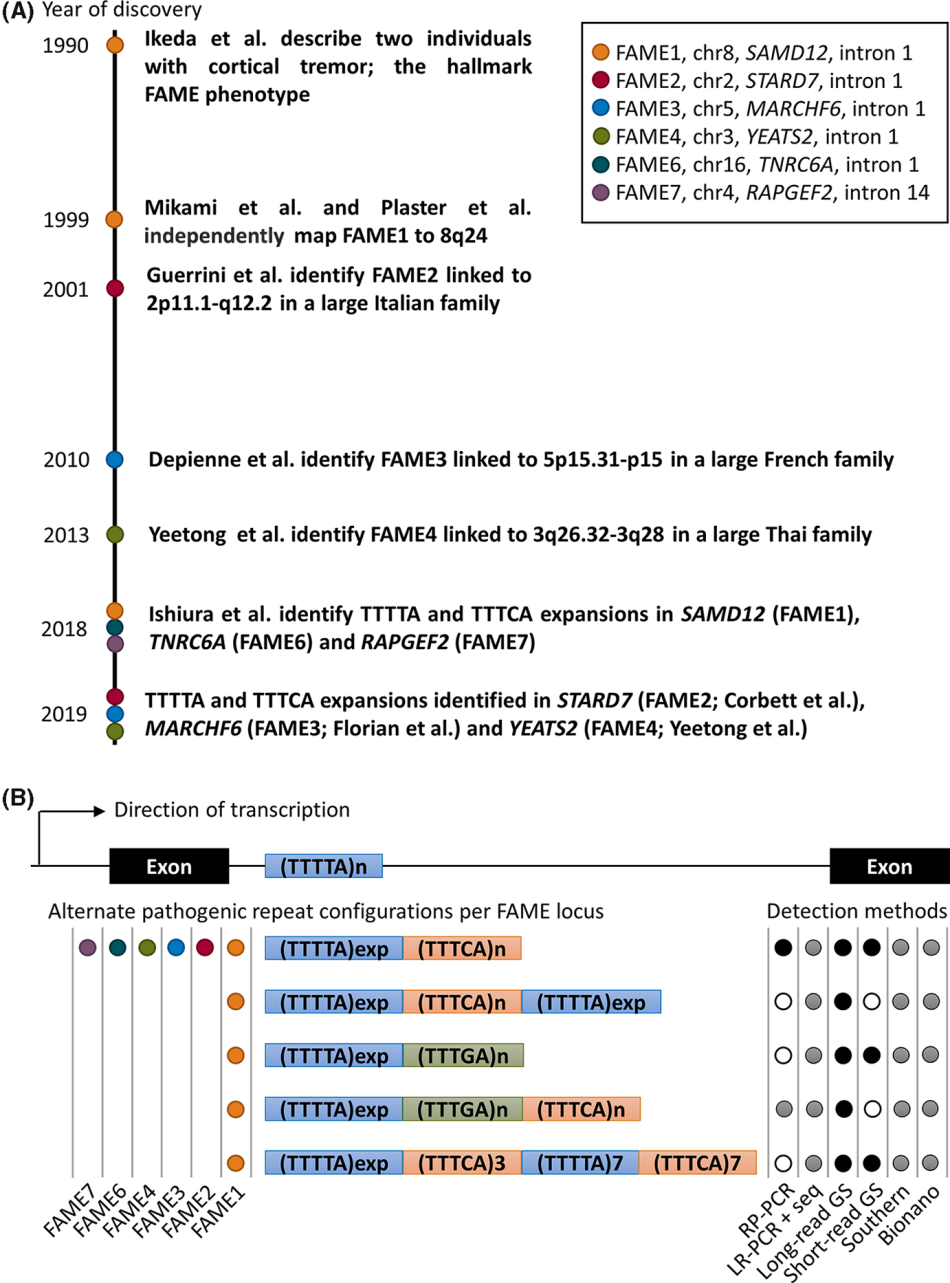
History of familial adult onset myoclonus epilepsy (FAME) genetics and methods for detecting FAME repeat expansions. (A) Timeline depicting years in which key events leading to the discovery of the TTTTA and inserted TTTCA repeat expansions occurred. Colored points on the time line map to events corresponding to particular FAME loci as per the supplied key in the figure. (B) A stylized “FAME gene” indicating the intronic location of the wild‐type TTTTA motif between two exons. For the majority of FAME genes, the expansion occurs in the first intron, except for *RAPGEF2* corresponding to FAME7, in which the expansion is in intron 14. Below the stylized gene are representations of the consensus repeat expansion motif configurations that have been reported. Colored dots to the left of each repeat configuration indicate that the configuration has been detected at least once at that FAME locus. To the right of each configuration, the black, gray, or white dots indicate the applicability of the detection method to that configuration. Black dots indicate the method is suitable, gray dots indicate that in some circumstances the method may produce a false negative or false positive result, and white dots indicate the method is highly likely to produce a false negative result. Bionano, Bionano optical genome mapping; GS, genome sequencing; LR‐PCR + seq, long‐range PCR plus sequencing; PCR, polymerase chain reaction; RP‐PCR, repeat‐primed PCR; Southern, Southern blot.

By initially targeting known genetic loci associated with epilepsy at the time, Mikami et al.^3^ serendipitously identified a positive hit on chromosome 8 segregating with FAME in a large Japanese family (Online Mendelian Inheritance in Man [OMIM], FAME1: 601068). They refined the localization of the gene for FAME1 to 8q23.3–q24.11 (Figure 1A).^3^ This interval does not include the *SAMD12* gene now known to harbor the TTTTA and TTTCA repeat expansions that cause FAME1.^4^ In hindsight and based on the remapping of this family,^5^ a probable genotyping error in a single individual in the pedigree (II‐18) defined the telomeric end of the linkage interval, which would otherwise have included *SAMD12*.^3^ From a genome‐wide linkage analysis of four Japanese families, Plaster et al.^6^ also localized FAME1 to 8q24 (Figure 1A). Based on the knowledge of the position of markers on chromosome 8 at the time, their reported interval also did not include the *SAMD12* gene.^6^ Reanalysis of these data by Cen et al.^7^ 15 years later, with the benefit of an available human genome sequence, reassigned the Plaster et al. interval such that it overlapped with intervals identified in other linkage studies and included *SAMD12*. Three other FAME1 linkage studies involving either Japanese or Chinese families living with FAME followed, which in combination narrowed the chromosome 8 interval to only 4.17 Mbp.^5^, ^7^, ^8^

Guerrini et al.^9^ linked autosomal dominant cortical myoclonic epilepsy (ADCME) in a large pedigree from Northern Italy covering approximately 21.3 Mbp and spanning the centromere on chromosome 2 (Figure 1A). Several linkage studies involving both Northern and Southern Italian families, with individuals who were phenotypically identical to FAME1, also mapped to the chromosome 2 ADCME interval, strongly suggesting ADCME and FAME1 were variants of the same epilepsy syndrome but mapping to different genes (OMIM, FAME2: 607876).^10^, ^11^, ^12^ Madia et al.^13^ provided strong evidence for a founder effect in all Italian FAME2 families and narrowed the chromosome 2 interval to 17.1 Mbp. Similar to FAME1, successive linkage studies in a Spanish,^14^ an Italian,^15^ and a New Zealander/Australian family of European ancestry^16^ progressively reduced the boundaries of the FAME2 linkage interval. In a novel approach that took advantage of the known FAME2 founder effect, Henden et al.^17^ combined genotyping data from many of the previously analyzed FAME2 families and used identity by descent mapping rather than traditional linkage to narrow the FAME2 interval to 9.78 Mbp.

Two additional FAME loci were discovered by linkage in the period prior to the discovery of the noncoding repeat expansions that cause all forms of FAME. FAME3 (OMIM: 613608) was mapped to a 9.31‐Mbp interval on chromosome 5 in French,^18^ Chinese,^19^, ^20^ and Dutch^21^ families and FAME4 (OMIM: 615127) was mapped to a 10‐Mpb interval on chromosome 3 in a large Thai family (Figure 1A).^22^

## 3 THE HUNT FOR THE GENETIC CAUSE OF FAME

A series of novel candidate variants were identified in plausible genes within the mapped FAME intervals. These included NM_173851.3:c.206A > T:p.(Tyr69Phe) in *SLC30A8* (FAME1),^7^ NM_000682.7:c.675_686delinsGTTTGGCAG in *ADRA2B* (FAME2),^23^ and NM_001332.4:c.3130G > A:p.(Glu1044Lys) in *CTNND2* (FAME3).^21^ Isolated variants, all of which fell outside known FAME linkage intervals, were also identified: NM_015902.6:c.5720G > A:p.(Arg1907His) in *UBR5* in a Chinese family,^24^ NM_003560.4:c.475G > A, p.Ala159Thr in *PLA2G6* in a Chinese family (who now have a confirmed *SAMD12* repeat expansion),^25^, ^26^ NM_138326.3:c.77G > A:p(Trp26*) in *ACMSD* in a Spanish family,^27^ and NM_003946.7:c.61G > C:p.(Glu21Gln) in *NOL3* in a Canadian family with familial cortical myoclonus without epilepsy.^28^ Finally, a homozygous variant, NM_005076.5:c.504del (p.Trp168fs) in *CNTN2*, segregated with autosomal recessive FAME (OMIM, FAME5: 615400) in an Egyptian family.^29^ All of these genes have failed to replicate with variants in other similarly mapped families, suggesting they were not the cause. Gene candidate sequencing approaches based on known gene functions (e.g., ion channels, known genes implicated in epilepsy), gene expression patterns, or gene coexpression networks were also unsuccessful.^14^, ^17^, ^18^ The strong suspicion based on the late onset of FAME and apparent absence of plausible coding candidate variants in short read exome and genome sequencing was that FAME was caused by either noncoding single nucleotide variants, structural variants, or repeat expansions.

By making a close inspection of single nucleotide polymorphism genotypes within the 4.17‐Mbp interval in six Japanese families, Ishiura et al.^4^ identified a shared haplotype of only 134 kbp covering exon 4 and part of intron 4 from the *SAMD12* gene. Apparent non‐Mendelian inheritance within a parent–child trio of a variable length TTTTA pentanucleotide repeat located in intron 4 of *SAMD12* led to closer inspection of this sequence. Genome sequencing data from an affected individual suggested the insertion of a nonreference TTTCA pentanucleotide repeat at the site of the TTTTA repeat and was the first evidence that the long‐sought noncoding variant causing FAME1 had been identified.^4^ In the same study, 47 FAME1 families were confirmed to have TTTTA and inserted TTTCA expansions in *SAMD12* and in two families novel loci were identified by discovery of noncoding TTTTA and inserted TTTCA expansions in *TNRC6A* located on16p21.1 (OMIM, FAME6: 618074) and *RAPGEF2* located on 4q32.1 (OMIM, FAME7: 618075), respectively.^4^ Within a year, similar noncoding TTTTA and inserted TTTCA repeat expansions were located in the linkage intervals for FAME2: *STARD7* intron 1,^30^ FAME3: *MARCHF6* intron 1,^31^ and FAME4: *YEATS2* intron 1 (Figure 1A).^32^ Individuals in >200 families have now had a confirmed genetic diagnosis of a TTTTA and inserted TTTCA repeat expansion within one of the FAME genes since the genetic cause of FAME1 was discovered. The pathogenic FAME1 repeat expansion has since been discovered in Chinese,^26^, ^33^, ^34^, ^35^, ^36^, ^37^, ^38^ Japanese,^39^ Indian,^40^, ^41^ Sri Lankan,^41^ and Thai^42^ families, all likely arising from one founder. The FAME1 repeat expansion was estimated to be approximately 16 800 years old and predicted to have arrived in Japan approximately 4300 years ago.^41^ Expansions within intron 14 of *RAPGEF2* of the recently discovered FAME7 locus have been identified in novel families from China and Japan.^35^, ^43^ There have been no additional genetic studies to date of FAME2, FAME3, FAME4, or FAME6 families since their initial discovery.

## 4 METHODS OF DETECTING FAME REPEAT EXPANSIONS

At present, there are no facilities that offer a specific molecular test for FAME; however, this could be rapidly addressed by adaptation of existing standard methodologies. Examples of detection methods for FAME repeats that could be adapted for genetic diagnosis of FAME include Southern blotting, repeat‐primed polymerase chain reaction (PCR), molecular combing, long‐range PCR combined with long‐ read sequencing, Cas9 endonuclease target enrichment‐based long‐read sequencing, and Bionano optical genome mapping (Figure 1B).^4^, ^30^, ^31^, ^43^, ^44^ FAME repeat expansions can be detected from short‐ or long‐read genome sequencing data, and this technique has been shown to have high sensitivity generally for detecting repeat expansion disorders in the setting of a clinical molecular genetics service.^4^, ^30^, ^31^, ^45^ Southern blotting, repeat‐primed PCR, and long‐range PCR are routinely used in genetic diagnosis of many repeat expansion disorders and are likely to be the most accessible technologies for established molecular pathology services. The specificity and sensitivity of the current methods for molecular diagnosis of FAME have not been quantified, due to incomplete knowledge about the repeat expansions, so a negative result should be considered with caution. Techniques that only assess the size of an expansion, such as Southern blotting, molecular combing, Bionano optical genome mapping, and long‐range PCR, must be combined with either repeat‐primed PCR or a sequencing technique to ensure the pathogenic TTTCA motif is present before a molecular diagnosis of FAME can be made. Despite having the advantages of requiring low amounts of genomic DNA input and low cost, long‐range PCR may produce a false negative result when the expanded allele is very large. Expansions composed only of TTTTA motifs exist at some loci in unaffected individuals and are not pathogenic. Alternate repeat configurations that contain either novel pentamer insertions (TTTGA), an inserted TTTCA expansion flanked by TTTTA expansions, or a very short TTTCA expansion usually do not provide a diagnostic result by repeat‐primed PCR (Figure 1B); therefore, alternative methods, ideally long‐read genome sequencing, should be applied where there is strong suspicion of FAME and a negative result is obtained with repeat‐primed PCR.^4^, ^26^, ^34^, ^44^, ^46^ Based on current knowledge, the highest diagnostic yields will be obtained for FAME1, and to a lesser extent FAME7 in patients of Asian ancestry^4^, ^35^, ^40^ and FAME2 and FAME3 in patients of European ancestry.^30^, ^31^ FAME4 and FAME6 have to date only been reported in single families of Asian ancestry.^4^, ^32^ One family with a FAME1 expansion and European ancestry has been reported; therefore, although ethnicity can guide triaging of tests, all loci should be considered when there is a strong clinical indication of FAME.^44^ Family history of FAME is the strongest indicator for a positive molecular diagnosis; very few diagnoses have been made from singleton families.^30^, ^39^

## 5 GENOTYPE TO PHENOTYPE RELATIONSHIPS IN FAME

Following the discovery of the TTTTA and inserted TTTCA expansions that cause all autosomal dominant forms of FAME, attention has turned to understanding genotype to phenotype relationships. There appear to be no major phenotypic distinctions between the different genetic forms of FAME. That the genes encompassing the intronic TTTTA and inserted TTTCA repeat expansions have different molecular functions and do not all operate in the same biochemical pathways suggests that the properties of the repeat and not the gene are the important driver of pathogenicity. Longer repeats or homozygosity for the repeats is correlated with an earlier age of disease onset and usually more severe neurological phenotypes.^4^, ^31^, ^35^, ^39^ However, it is not possible to make a meaningful prognosis for an individual patient based on the length of their repeat alone, and this suggests there are additional factors that influence symptom onset and severity. Florian et al.^31^ suggested that the TTTCA repeat content was more strongly correlated with onset of seizures in FAME3, whereas TTTTA content did not show the same correlation. Pan et al.^26^ suggested that individuals with a *SAMD12* repeat configuration in which the inserted TTTCA expansion is in between two TTTTA expansions have earlier symptom onset than individuals with the more frequently observed configuration in which the TTTTA expansion is adjacent to the TTTCA expansion. Mizuguchi et al.^43^ identified a huge degree of variability in the numbers of copies of TTTTA and TTTCA pentamers in FAME1 repeats between different individuals from different families, including in one individual where only 14 copies of the TTTCA motif were observed. To date, there is no evidence that TTTTA expansions alone are pathogenic.^26^

FAME TTTTA and inserted TTTCA repeat expansions exhibit both somatic and generational instability in their repeat copy numbers and structural arrangement of repeat motifs. Anticipation, where age of symptom onset decreases and disease severity increases over successive generations, is a common feature of most repeat expansion disorders and is correlated with intergenerational increases in repeat lengths. Anticipation occurs in FAME families; however, correlation of anticipation with an increase in repeat length between parent and child remains uncertain due to the low numbers of relationships where this has been studied at the molecular level.^31^, ^39^, ^46^ Paternal or maternal inheritance and maternal age have been investigated as potential risk factors; however, these have not shown significant correlation with germline repeat instability to date.^4^, ^26^, ^39^ There are no known genetic modifiers for susceptibility to germline or somatic repeat instability of FAME repeat expansions. A significant enrichment of longer TTTTA repeat alleles in the *TNRC6A* gene in individuals with FAME1 *SAMD12* repeat expansions compared to an unaffected control population has been noted; however, the reason for this remains unclear.^39^

Somatic variation of TTTTA and inserted TTTCA repeat expansions has been observed in FAME1, FAME2, and FAME3 both within one tissue and between different tissues from one individual.^4^, ^30^, ^31^ Somatic variability increases with the size of the expansions, with expansions larger than 10 kb showing multiple sizes and configurations within a single tissue.^31^ Given that most genotype–phenotype correlation studies to date used genomic DNA extracted from patient blood, repeat lengths and repeat configurations reported may not reflect the dominant repeat configuration in the brain of each individual.

## 6 SIGNIFICANCE OF THE DISCOVERY OF FAME REPEAT EXPANSIONS AND FUTURE DIRECTIONS

Unlocking the genetic cause of FAME was a breakthrough discovery in human disease genetics. The discovery places FAME broadly within the family of >50 repeat expansion disorders.^47^ Within these, FAME now accounts for the majority of a special category of transcribed but noncoding adenine and thymine (AT)‐rich pentamer expansions. There are now multiple options available to provide individuals with a genetic diagnosis based on the detection of TTTCA repeat expansions at any of the currently known loci, providing greater certainty than a test based on linkage, but these are yet to be translated to molecular pathology services beyond the sphere of laboratories specializing in FAME genetics research. There remain families in which known loci were excluded by linkage, or direct molecular tests for the known repeat expansions, suggesting additional FAME genes are yet to be discovered.^28^, ^39^, ^43^, ^48^, ^49^, ^50^ The questions of what modifies the correlation between repeat length and disease onset and which factors influence germline and somatic instability also remain open and will require new cell and animal preclinical models to uncover.^51^

## AUTHOR CONTRIBUTIONS

Mark A. Corbett drafted the original manuscript. All remaining authors contributed revisions and had intellectual input into the final manuscript.

## CONFLICT OF INTEREST STATEMENT

The authors have no conflicts of interest to declare. We confirm that we have read the Journal's position on issues involved in ethical publication and affirm that this report is consistent with those guidelines.
